# Medial maxillary (nasomaxillary buttress) fracture: Two case reports

**DOI:** 10.1097/MD.0000000000031845

**Published:** 2022-11-18

**Authors:** Masaru Ogawa, Satoshi Yokoo, Hidenori Nakamura, Takahiro Yamaguchi, Keisuke Suzuki, Yu Takayama, Takaya Makiguchi

**Affiliations:** a Department of Oral and Maxillofacial Surgery, and Plastic Surgery, Gunma University Graduate School of Medicine, Showa-machi, Maebashi City, Gunma, Japan.

**Keywords:** medial maxillary fracture, nasal fracture, nasomaxillary buttress fracture, NOE fracture, zygomatic fracture

## Abstract

Medial maxillary fractures are defined as fractures of the medial aspect of the maxilla, that is, fractures of the nasomaxillary buttress. The diagnosis of medial maxillary (nasomaxillary buttress) fractures is challenging because of its similarities with nasal, and naso-orbito-ethmoidal (NOE) fractures.

**Patient concern:**

The patients included a 17-year-old Japanese man and a 37-year-old Japanese woman with facial injuries. Swelling of the affected cheek, nasal hemorrhage, periorbital ecchymosis, and C-shaped deviation of the nasal dorsum with normal nasal tip and root position were observed. Mild hypesthesia was noted in the affected region innervated by the left infraorbital nerve. Telecanthus, double vision, eye movement disorder, epiphora, trismus, tooth fracture, and malocclusion were absent in both cases.

**Diagnosis:**

Based on clinical signs and imaging findings, the patients were diagnosed with medial maxillary (nasomaxillary buttress) fracture.

**Intervention:**

The 17-year-old man underwent a reduction of the medial aspect of the maxillary bone with the treatment of concomitant fractures, and the 37-year-old woman did not request treatment.

**Lessons:**

Medial maxillary (nasomaxillary buttress) fractures are not included in any current classification of the maxillary fracture patterns. As medial maxillary (nasomaxillary buttress) fractures are similar to nasal, and NOE fractures, careful differential diagnosis is required before selecting the surgical procedure to be performed because the treatment of concomitant fractures is also necessary.

## 1. Introduction

Four primary processes such as frontal, palatine, zygomatic, and alveolar processes form a thick portion of the maxillary bone, and the thicker portion forms a buttress extending from the maxillary alveolar process to the frontal cranium. Three vertical buttresses are present in each part of the maxillary bone which increases the strength of the midface to protect the cranium. Medial maxillary fractures are defined as fractures of the medial aspect of the maxilla, that is, fractures of the nasomaxillary buttress. Medial maxillary (nasomaxillary buttress) fracture was initially described by May et al^[1]^ in 1973. In typical cases, depression of the lateral wall of the nasal cavity morphologically results in a C-shaped deviation of the nasal dorsum, with narrowing of the nasal cavity, nasal obstruction, and nasal hemorrhage as symptoms. However, different symptoms manifest corresponding to the region to which the fracture extends because the medial surface of the maxillary bone joins with other bones, including the nasal, ethmoid, and lacrimal bones. If a medial maxillary (nasomaxillary buttress) fractures extend toward the frontal process of the maxilla, the medial orbital wall including the lacrimal and ethmoid bones, and the nasal bone are affected. If the fracture extends continuously to the orbital floor, a blow-out fracture develops. Surgical treatment of medial maxillary (nasolabial buttress) fractures requires an accurate diagnosis and careful planning in each case because the treatment of concomitant fractures is also necessary. Recently, Yoshioka et al^[2]^ and Hwang et al^[3]^ have only reported 8 and 15 cases of this fracture, respectively. Medial maxillary (nasomaxillary) fractures are not included in any current classification of the maxillary fracture patterns, which include Le Fort-type, sagittal, or fracture of the alveolar process. Therefore, the clinical conditions and diagnosis of medial maxillary (nasomaxillary buttress) fractures are still not understood by many surgeons and radiologists. We present 2 cases of medial maxillary (nasomaxillary buttress) fracture in terms of differential diagnosis and pathogenesis.

## 2. Case reports

### 2.1. Patient 1

**Figure 1.**
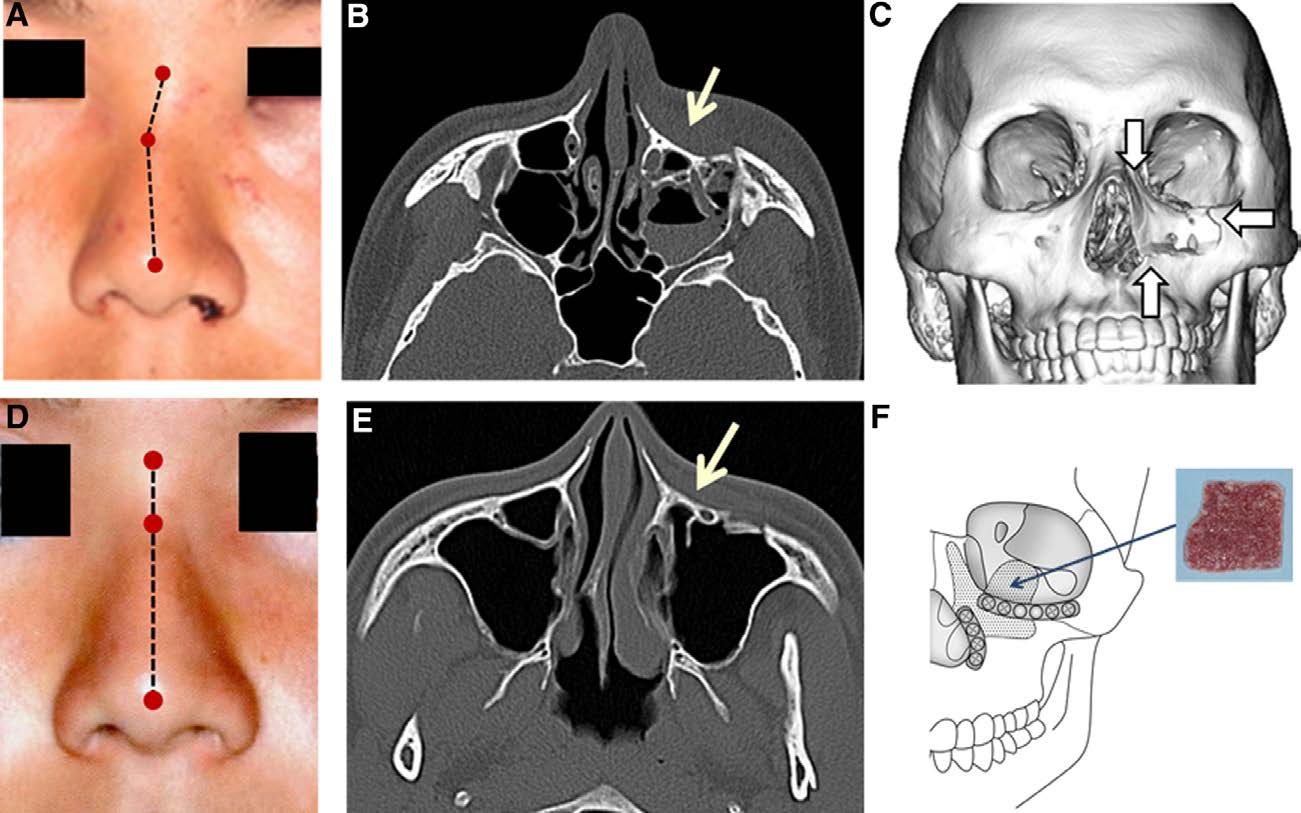
Patient 1. (A) C-shaped deviation of the nasal dorsum. (B) The horizontal view on CT. (C) 3D-CT. (D) The deviated nasal dorsum is improved. (E) The deviated fragment is reduced at one year postoperatively. (F) A sheet prepared with cancellous bone from the anterior superior iliac crest is transplanted. For fixation of the orbital rim and border of the piriform aperture, an absorbable plate is used. 3D = three-dimensional, CT = computed tomography.

A 17-year-old Japanese man was bruised on his face by a hard baseball and visited the Department of Oral and Maxillofacial Surgery, Gunma University Hospital. Swelling of the left cheek and nasal hemorrhage were observed. C-shaped deviation of the nasal dorsum was observed, but the tip and root of the nose remained in a fairly normal position. Mild hypesthesia was noted in the region innervated by the left infraorbital nerve. Telecanthus, double vision, eye movement disorder, epiphora, trismus, tooth fracture, and malocclusion were absent. In the horizontal view on computed tomography (CT), a fracture line was noted in the anterior wall of the maxilla and the bone fragment was depressed. The medial aspect of the maxillary bone (from the border of the pyriform aperture to the frontal process of the maxilla and orbital rim) deviated inside and medially on three-dimensional (3D)-CT. A fracture of the maxillary bone region constituting the orbital floor was also observed. We had a definitive diagnosis of a compound fracture of the left mesial maxillary (nasomaxillary buttress) and blow-out fracture. As the reduction of the lateral nasomaxillary buttress by closed reduction from the nasal cavity alone was difficult, open reduction was performed under general anesthesia, through a subciliary incision and an oral vestibular incision. Reduction of the medial aspect of the maxillary bone was performed by inserting an elevator into the maxillary sinus. For the fixation of the orbital rim and border of the pyriform aperture, an absorbable plate was used. For the bone defect of the orbital floor formed by the deviation of the medial aspect of the maxillary bone and blow-out fracture, a sheet prepared with only cancellous bone from the anterior superior iliac crest was transplanted. The nasal dorsum improved aesthetically, and the disorder of the inferior orbital nerve had resolved at the 1-year postoperative follow-up (Fig. 1).

### 2.2. Patient 2

**Figure 2.**
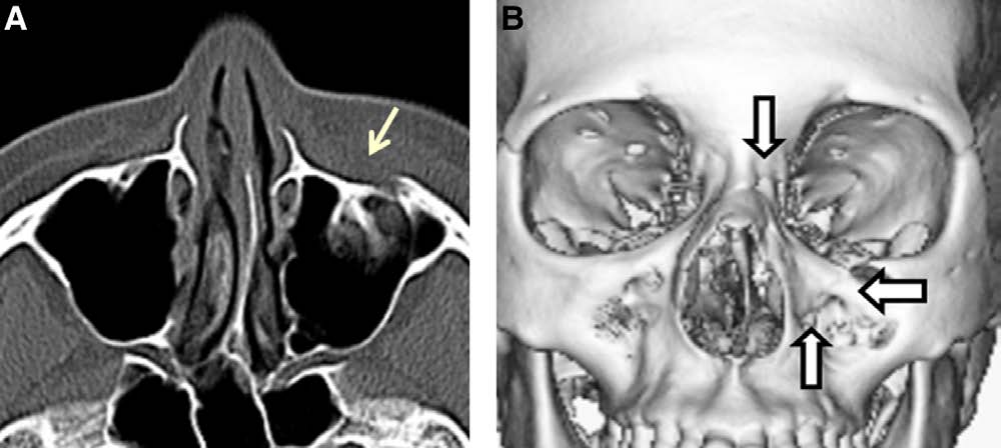
Patient 2. (A) In the horizontal view on CT, a fracture line is noted in the anterior wall of the maxilla and the bone fragment is depressed. (B) The medial aspect of the maxillary bone deviates inside and medially on 3D-CT. 3D = three-dimensional, CT = computed tomography.

A 37-year-old Japanese woman hit the left side of her face on the edge of a desk during a fall. Swelling of the left cheek, nasal hemorrhage, periorbital ecchymosis, and C-shaped deviation of the nasal with normal nasal tip and root position were observed. Hypoesthesia was noted in the region innervated by the left infraorbital nerve. Telecanthus, double vision, eye movement disorder, epiphora, trismus, tooth fracture, and malocclusion were absent. In the horizontal view on CT, a fracture line was noted in the anterior wall of the maxilla, and the bone fragment was slightly depressed. The medial aspect of the maxillary bone slightly deviated inside and medially on 3D-CT. We had a definitive diagnosis of left mesial maxillary (nasomaxillary buttress) fracture. Surgery was not performed because the patient did not request a treatment (Fig. 2).

## 3. Discussion

Medial maxillary (nasomaxillary buttress) fractures are characterized by a C-shaped deviation of the nasal dorsum.^[4]^ However, this fracture cannot be diagnosed based on this deviation alone because a similar deviation is present in simple nasal fractures and NOE fractures. In medial maxillary (nasomaxillary buttress) fractures, the tip and root of the nose remain in a fairly normal position because the nasal bones are not disturbed.

**Figure 3.**
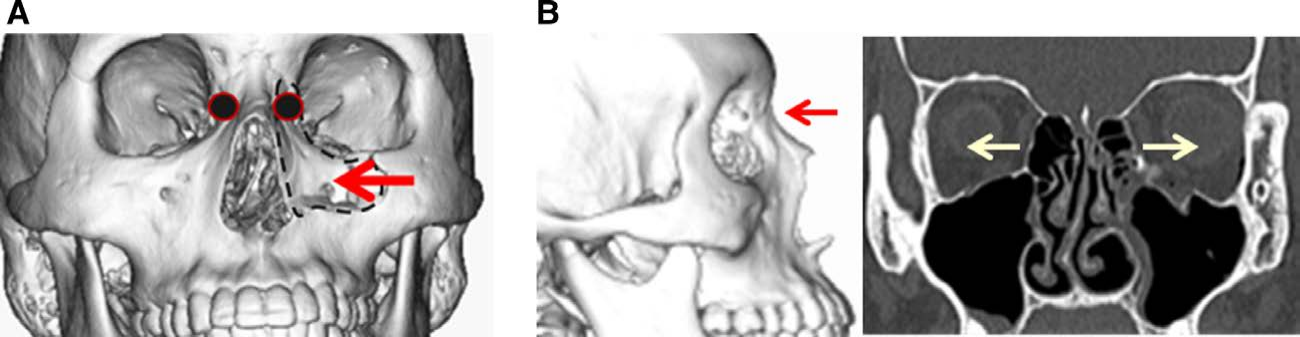
(A) Separation of the medial canthus does not occur in medial maxillary (nasomaxillary buttress) fracture because a ligament-attached bone fragment (●) is deviated inside and/or toward the middle with the maxillary frontal process due to an external lateral force. (B) In NOE fracture, separation of the medial canthus occurs because of an external force from the front. NOE = naso-orbito-ethmoidal.

A NOE fracture adds signs of lacrimal duct obstruction and separation of the medial canthus (telecanthus). Theoretically, telecanthus observed in NOE fractures is a possible complication due to the deviation of the frontal process of the maxillary bone. However, telecanthus was not noted in any of the medial maxillary (nasomaxillary buttress) fractures reported by May et al,^[1]^ Yoshioka et al,^[2]^ Anderson et al,^[4]^ and Hillstrom et al,^[5]^ or in the patients described in this report. May et al^[1]^ and Hillstrom et al^[5]^ suggested that the difference in the fracture pattern between NOE and medial maxillary (nasomaxillary buttress) fractures is due to the difference in the direction of the external force. Medial maxillary (nasomaxillary buttress) fractures were suggested to occur through an external force on the lateral surface of the medial maxillary wall, that is, an external force from the lateral side in many cases. Telecanthus is unlikely in this situation because the medial canthal tendon deviates medially and/or toward the midline with the frontal process of the maxillary bone. The 2 patients described in this report were also injured by an external force from the lateral side. On the other hand, Trost et al^[6]^ proposed that the difference in the level of the injured region is observed as a difference in the fracture pattern of medial maxillary (nasomaxillary buttress) fractures compared to nasal and NOE fractures; the point of impact in medial maxillary (nasomaxillary buttress) fractures are lower, at the limit between the nasal pyramid and the medial part of the infraorbital rim. In contrast, in nasal or NOE fractures in the Markowitz classification,^[7]^ the point of impact is higher on the nasal pyramid, leading to the impaction of the nasal pyramid in the ethmoidal cells and frontal sinus. This difference in the level of the injured region is also considered to be related to the development of telecanthus (Fig. 3).

In conclusion, medial maxillary (nasomaxillary buttress) fractures are not included in any current classification of the maxillary fracture patterns. As medial maxillary (nasomaxillary buttress) fractures are similar to nasal and NOE fractures in clinical signs, careful differential diagnosis is required before selecting the surgical procedure to be performed.

## Acknowledgements

We would like to thank Editage (www.editage.com) for English language editing.

## Author contributions

MO and SY drafted the manuscript as corresponding authors. MO, SY, and TM performed the clinical diagnosis and the operation. HN and TM managed the patients. MO, SY, TY, KS, and YT participated in the operation.

**Formal analysis:** Masaru Ogawa.

**Project administration:** Masaru Ogawa, Satoshi Yokoo.

**Writing – original draft**: Masaru Ogawa.

**Writing – review & editing:** Hidenori Nakamura, Takahiro Yamaguchi, Keisuke Suzuki, Yu Takayama, Takaya Makiguchi.

All authors read and approved the final manuscript.
